# Coral Reefs and People in a High-CO_2_ World: Where Can Science Make a Difference to People?

**DOI:** 10.1371/journal.pone.0164699

**Published:** 2016-11-09

**Authors:** Linwood Pendleton, Adrien Comte, Chris Langdon, Julia A. Ekstrom, Sarah R. Cooley, Lisa Suatoni, Michael W. Beck, Luke M. Brander, Lauretta Burke, Josh E. Cinner, Carolyn Doherty, Peter E. T. Edwards, Dwight Gledhill, Li-Qing Jiang, Ruben J. van Hooidonk, Louise Teh, George G. Waldbusser, Jessica Ritter

**Affiliations:** 1 Université de Bretagne Occidentale, UMR6308 AMURE, IUEM, Plouzané, France; 2 Duke University, Durham, North Carolina, United States of America; 3 RSMAS/MBE, University of Miami, Miami, Florida, United States of America; 4 University of California Davis, Policy Institute for Energy, Environment and the Economy, Davis, California, United States of America; 5 Ocean Conservancy, Washington, D.C., United States of America; 6 Natural Resources Defense Council, New York, New York, United States of America; 7 The Nature Conservancy and the University of California, Santa Cruz, Ocean Sciences, Santa Cruz, California, United States of America; 8 Institute for Environmental Studies, VU University, Amsterdam, The Netherlands; 9 World Resources Institute, Washington, D.C., United States of America; 10 James Cook University, ARC Centre of Excellence for Coral Reef Studies, Townsville, Australia; 11 Coral Reef Conservation Program, NOAA, Silver Spring, Maryland, United States of America; 12 Ocean Acidification Program, NOAA, Silver Spring, Maryland, United States of America; 13 Cooperative Institute for Climate and Satellites, Earth System Science Interdisciplinary Center, University of Maryland, College Park, Maryland, United States of America; 14 NOAA Atlantic Oceanographic and Meteorological Laboratory, Ocean Chemistry and Ecosystems Division, Miami, Florida, United States of America; 15 Cooperative Institute for Marine and Atmospheric Studies, Rosenstiel School of Marine and Atmospheric Science, University of Miami, Miami, Florida, United States of America; 16 Institute for Oceans and Fisheries, The University of British Columbia, Vancouver, British Columbia, Canada; 17 Oregon State University, College of Earth, Ocean, and Atmospheric Sciences, Corvallis, Oregon, United States of America; 18 National Wildlife Foundation, Washington, D.C., United States of America; University of Bologna, ITALY

## Abstract

**Reefs and People at Risk:**

Increasing levels of carbon dioxide in the atmosphere put shallow, warm-water coral reef ecosystems, and the people who depend upon them at risk from two key global environmental stresses: 1) elevated sea surface temperature (that can cause coral bleaching and related mortality), and 2) ocean acidification. These global stressors: cannot be avoided by local management, compound local stressors, and hasten the loss of ecosystem services. Impacts to people will be most grave where a) human dependence on coral reef ecosystems is high, b) sea surface temperature reaches critical levels soonest, and c) ocean acidification levels are most severe. Where these elements align, swift action will be needed to protect people’s lives and livelihoods, but such action must be informed by data and science.

**An Indicator Approach:**

Designing policies to offset potential harm to coral reef ecosystems and people requires a better understanding of where CO_2_-related global environmental stresses could cause the most severe impacts. Mapping indicators has been proposed as a way of combining natural and social science data to identify policy actions even when the needed science is relatively nascent. To identify where people are at risk and where more science is needed, we map indicators of biological, physical and social science factors to understand how human dependence on coral reef ecosystems will be affected by globally-driven threats to corals expected in a high-CO_2_ world. Western Mexico, Micronesia, Indonesia and parts of Australia have high human dependence and will likely face severe combined threats. As a region, Southeast Asia is particularly at risk. Many of the countries most dependent upon coral reef ecosystems are places for which we have the least robust data on ocean acidification. These areas require new data and interdisciplinary scientific research to help coral reef-dependent human communities better prepare for a high CO_2_ world.

## Introduction

Hundreds of millions of people worldwide depend on coral reef ecosystems[1]. Coral reef ecosystems create natural barriers that protect shorelines from storm surge and erosion—defending villages, businesses, and coastal residents[2]. Coral reef ecosystems also support fisheries that provide food [3], jobs, and income for local communities [4,5] as well as tourism and recreation that contribute to jobs, profits, taxes, and foreign income[3]. The recreational and cultural services provided by these ecosystems also benefit local communities and people.

**Fig 1:**
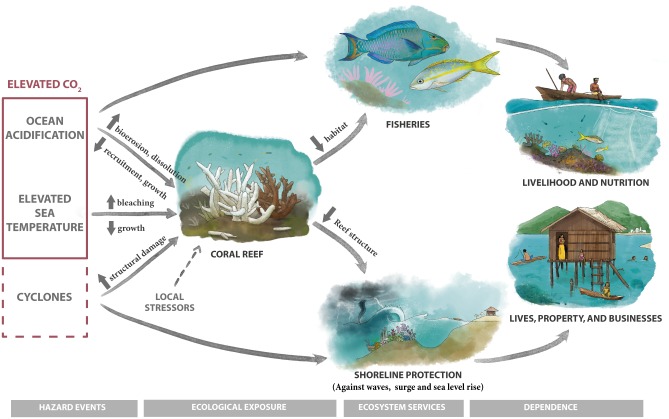
A conceptual diagram linking stresses related to increased atmospheric CO_2_ (elevated sea surface temperature and ocean acidification), storms, and local stressors to coral reef condition, selected ecosystem services provided by reefs, and human dependence on these ecosystem services. Solid lines represent relationships evaluated in this study.

Increasing levels of carbon dioxide in the atmosphere put shallow, warm-water coral reef ecosystems, and the people who depend upon them at risk from two key global environmental stresses: 1) elevated sea surface temperature (that can cause coral bleaching and related mortality), and 2) ocean acidification (OA). Bleaching and OA can compound local reef stresses that will hasten the loss of the ecosystem services provided by reefs (Fig 1). Structural damage to coral reefs can result in more severe coastal inundation that puts lives and property at risk [6]. These environmental stresses will also decrease coral ecosystem health and productivity [7,8], which in turn could jeopardize nutrition, livelihoods, and local incomes that depend on reef fisheries and could impact reef-related tourism[5]. We acknowledge that coral reef ecosystems are also threatened by other local stressors that include overfishing, destructive fishing, disease, predators, pollution, eutrophication, sedimentation, and episodic de-oxygenation [9]. Nevertheless, we focus on elevated sea surface temperature and OA because these factors are largely beyond the control of coastal communities, managers of marine protected areas, and other management bodies that exist at the country level or smaller [10]. Coral reef countries have four primary options to counter the threats to reefs caused by the emission of CO_2_ [11]: 1) urge governments of major CO_2_-emitting nations (many of which are also home to coral reefs) to reduce carbon emissions that cause both climate change and OA, 2) reduce damages to corals caused by local environmental stressors that can make these problems worse, and 3) improve and/or restore associated ecosystems (e.g. mangroves) to a state that could replace lost ecosystem services and thus minimize impacts on people. Engineering responses, other than green infrastructure and restoration, to counter these global threats have also been proposed [12,13], but they are largely untested. Without these measures, countries dependent on coral reef ecosystems may need to cope with a world with greatly diminished coral reefs–a response that could spur human migration.

The ecological and social impacts of CO_2_-related threats to reefs will not be the same across the globe[4,5,14]. Increasing levels of atmospheric CO_2_ will cause the most immediate and serious problems where a) human dependence on coral reef ecosystems is high, b) sea surface temperature reaches critical levels soonest, and c) OA levels are most severe. Where these elements align, swift action will be needed to protect people’s lives and livelihoods, but such policy action must be informed by data and science. Correspondingly, places where the threats of OA and climate change are low may serve as potential refugia for coral reef organisms and larvae.

Sufficient indicator data exist to create preliminary maps of the potential threats to coral reef ecosystems posed by a high-CO_2_ world and the people and countries that will be affected. Two previous studies examined the combined threats faced by coral reefs from local and global stressors as well as an array of human dimensions that include human dependence and adaptive capacity [15,1]. We update and build on previous studies by developing an indicator analysis that focuses specifically on the threats to coral reefs and people from a high-CO_2_ future. Our analysis also differs from previous studies in that we focus on the dependence of people on coral reef ecosystem services without attempting to assess their adaptive capacity. Adaptive capacity, while an important factor in evaluating vulnerability and risk within a region, is often represented with metrics that do not accurately convey conditions of coastal communities [16]. By focusing on fewer dimensions, we are able to: leverage data that allows us to increase the granularity of the analysis, increase the transparency of the analysis, and improve our ability to link high-CO_2_ threats to human outcomes.

There still is much to be learned about the impacts of global change on coral reef ecosystems and the people who depend on them. We need to better understand the ecological science regarding how coral reefs are affected by both global and local environmental stressors and how people, in turn, respond to these changes. Social and economic factors should be directly considered in setting research priorities about locations for new science. An examination of current human dependence on coral reef ecosystems and our current state of knowledge about the large scale and unavoidable threats that will result from increasing concentrations of CO_2_ in the atmosphere can help us identify where new science and data are needed to help people deal with these environmental changes and coral reef decline.

### Why sea surface temperature and ocean acidification matter

Coral bleaching, mortality, and disease caused by elevated sea surface temperature, have direct impacts on coral reef ecosystems [8]. Sustained bleaching events can cause coral reef death [17]. Historically, the time between mass mortality events allowed coral reef ecosystems to recover from the damage caused by coral bleaching as new coral larvae could settle and grow in damaged areas. As these mortality events become more frequent, it is harder for coral reef ecosystems to recover. Coral bleaching has been shown to damage coral reef ecosystems [18,19] and can lead to bioerosion if corals die, eventually leading to the loss of reef height and structural complexity, also known as rugosity [20]. Reef structure provides shoreline protection [6,21]. Ferrario et al. [22] found that coral reefs can dissipate approximately 97% of wave energy. The reef crest is the most important attenuation factor, contributing to 86% of wave attenuation. Roughness or rugosity is the next most important attenuation factor [22]. Moreover, the three-dimensional structure of coral reefs also provide habitat for reef fish and other organisms that support the livelihoods of coastal areas [23]. To maintain these services, reefs must not only maintain their structure, but must keep pace with sea level rise.

The ability of coral reef ecosystems to recover from damaging events is likely to be suppressed by the elevated sea surface temperature and OA expected to occur in a high-CO_2_ world. Van Hooidonk et al. [24,25] used projections under the Intergovernmental Panel on Climate Change’s RCP8.5 emissions scenario to show the potential spatial distribution of sustained, future high sea surface temperatures measured as the year when an area experiences at least 8 degrees Celsius degree-heating weeks (DHW) annually. A degree heating week is a standard measure of heat accumulation over the previous twelve weeks and represents the number of weeks an area has experienced temperatures in excess of 1 degree Celsius above the highest mean summer time temperature. Here we use this same threshold of 8 DHWs to indicate where future increases in sea surface temperature will lead to sustained bleaching and a high likelihood of coral mortality. Changes in ocean carbonate chemistry due to increasing atmospheric CO_2_, known as OA and often measured by aragonite saturation state (Ω_ar_), also poses a severe threat to corals and reef ecosystems[4]. While much of the research focus, and debate, to date has been on the role of OA in the reduction of calcification rates on coral reefs [26–28], OA can significantly impair other ecological and physiological functions. For instance, coral larval success may be impaired at much more modest levels of OA. Ω_ar_ levels of 3.1 or less, a level some coral reefs will experience in the next decade, are known to impair larval recruitment of some corals[29,30]. Similar levels of OA can also reduce growth rates in some corals [31]. Experimental evidence shows that increased OA and thermal stress combined have a greater harmful effect on both larval success and growth rates than either factor alone [32], which could make coral recovery even more difficult when both stressors occur simultaneously (and at less severe levels than those required to induce harm by either stressor alone). Additionally, a variety of other coral reef organisms have also been shown to suffer from thermal stress and OA [7,33].

## Materials and Methods

### Where are the greatest potential risks to reefs and people in a high-CO_2_ world?

We use an indicator approach to identify places where key environmental factors driven by a high-CO_2_ world may put coral reef-dependent people most at risk [34]. Mapping indicators has been proposed as a way of “integrating natural and social sciences to identify actions and other opportunities while policy, stakeholders and scientists are still in relatively early stages of developing research plans” to combat global environmental change [33]. As such, an indicator approach allows for a focus on a spatial understanding of key characteristics of the social-ecological system, even in the absence of a complete set of science and data that would be needed to create more complex models of ecological processes and people’s responses to change in ecosystem conditions.

To identify where people are at risk from CO_2_-driven threats to coral reefs, we map indicators of two key aspects of current human dependence on coral reefs (people who benefit from the shoreline protection provided by reefs and reef-related fisheries) and two key indicators of oceanic change in a high CO_2_ world (the onset of high thermal stress in terms of the year that sea surface temperature reaches 8 DHW annually [24,25] and the expected level of OA in year 2050). Recent studies show that the precise role that increased sea surface temperature and OA have on coral reef ecosystem conditions and health is complicated [27,35,36] and may vary regionally [37]. With that in mind, these indicators are not intended to be predictive of coral reef death. Instead, we use an indicator approach to reflect the spatial distribution of the intensity of environmental stress on corals that could result from increased levels of atmospheric CO_2_ that are projected to occur during the twenty first century, if emissions continue under assumptions of business as usual [38].

**Fig 2:**
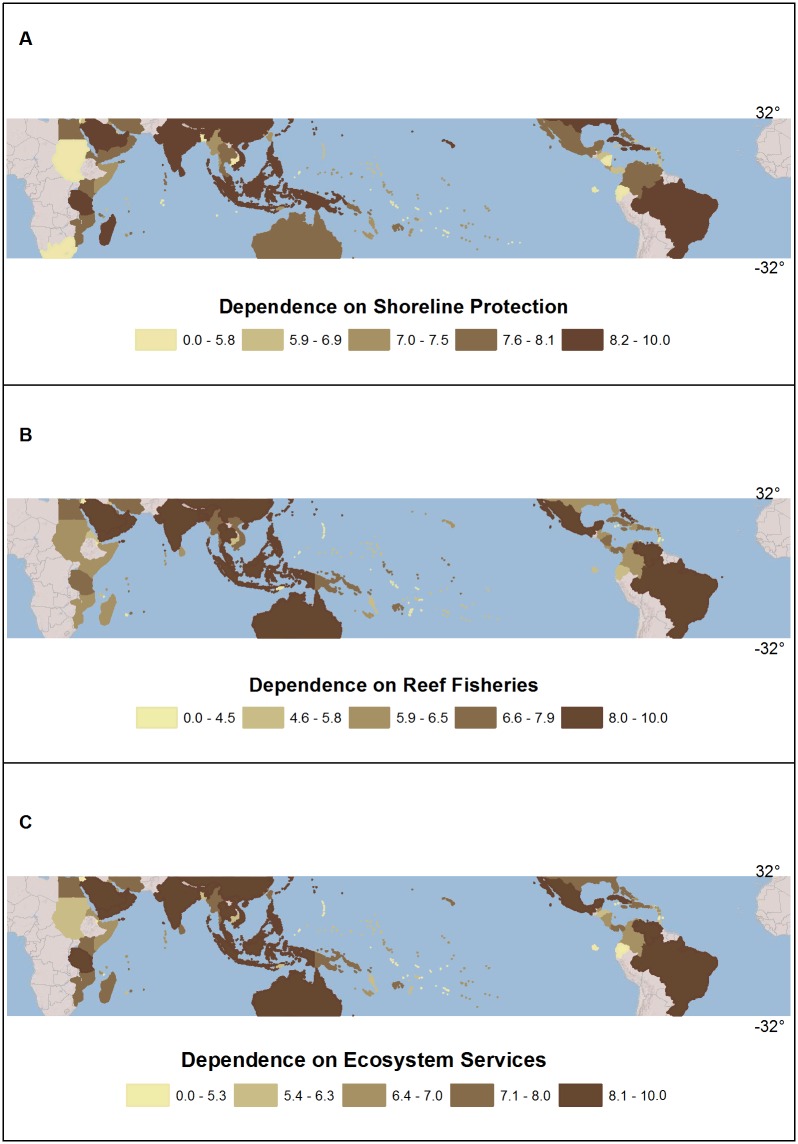
Scores of human dependence on coral reef ecosystem services, by country. Panel A provides the normalized scores for human dependence on shoreline protection, Panel B shows the normalized scores for dependence on reef fisheries, and Panel C shows combined human dependence. All scores are normalized on a scale from 0–10. Higher scores reflect higher human dependence. Countries are binned by quintile in the legend.

We score and map two indicators of human dependence on coral reefs at the country level: shoreline protection and coral reef fisheries. We map human dependence at the country level so that our results are commensurate with similar country-level studies of coral reef vulnerability conducted at a global scale [15,1,39]. To score the relative human dependence of each country in terms of the shoreline protection provided by reefs, we use calculations from Reefs at Risk Revisited [40] of the number of people in 2007 who lived at less than ten meters above sea level [41], near a shoreline that is within 3km of a coral reef and up to 4km inland (Table 1). For each country, we create a normalized score of the people at risk by taking the Z-score of lg(number of people) and rescaling [42] this from 0 to 10 (Table 1). To score and map the reliance of countries on coral reef fisheries, we use data from Teh et al. 2013 [40] based on the Sea Around Us project which estimates two components of reef-dependent fisheries landings in 2005: jobs and value (Table 1). Using the Sea Around Us data, we create similar normalized scores of lg(jobs) (Z-score re-scaled from 0–10) associated with reef fisheries and the estimated value of coral reef harvests (in real 2005 USD). (Value is also highly correlated with landed weight of reef fish, r = 0.86). To create a summary score of human dependence, we take the average score of: a) shoreline protection, and b) the higher score of reef fish jobs or value and renormalize it to obtain a score of 0–10 (Fig 2). We use only one score for fisheries dependence (the higher) in order to equally weight shoreline protection and fisheries dependence. These estimates are not projected into the future.

**Table 1:** Raw data and results of the normalized scoring for human dependence, by country (only countries for which data are available are shown). Ocean Provinces: Brazilian (B), Caribbean (C), Central Pacific (CP), Great Barrier Reef (GBR), Central Indian Ocean (CIO), Eastern Pacific (EP), Middle East (ME), Polynesia (P), South East Asia (SEA), Western Australia (WA), Western Indian Ocean (WIO).

Country	Population protected by coral reefs, # ofpeople (2007) [41]	# of Fishers involved in coral reef fisheries (2005) [40]	Value of reef fish harvest (2005, in real $) [40]	Normalized Score Population Protected (0–10)	Normalized Score Maximum of Fishers, Value (0–10)	Ocean Province
Aruba	70,982	1,018	331,268	6.8	4.4	C
Anguilla	4,174	208	788,289	5.1	5.0	C
United Arab Emirates	1,217,577	12,385	153,922,439	8.6	8.6	ME
American Samoa	33,296	1,847	121,901	6.4	3.7	CP, P
Antigua and Barbuda	24,649	2,134	10,621,547	6.2	6.8	C
Australia	316,027	29,593	467,219,756	7.7	9.4	GBR, SEA, WA
Bangladesh	1,318	230,498	N/A	4.4	8.0	SEA
Bahrain	575,191	7,200	63,046,838	8.1	8.0	ME
Bahamas	260,184	12,000	89,287,977	7.6	8.3	C
Belize	98,020	6,926	7,681,824	7.0	6.5	C
Bermuda	58,903	2,158	1,296,462	6.7	5.3	C
Brazil	1,239,637	144,433	180,174,864	8.6	8.8	B
Barbados	91,611	566	23,101	7.0	2.5	C
Brunei Darussalam	0	920	N/A	0	2.4	SEA
Cambodia	8,000	14,364	N/A	5.5	5.2	SEA
Cocos (Keeling) Islands	643	N/A	N/A	3.9	N/A	SEA
China	1,212,378	189,467	708,521,292	8.6	9.7	SEA
Cook Islands	13,919	3,971	430,936	5.8	4.5	CP, P
Colombia	345,743	12,188	4,930,352	7.8	6.2	C, EP
Comoros	334,444	12,077	N/A	7.8	5.0	WIO
Costa Rica	92,470	12,303	5,959,548	7.0	6.4	C, EP
Cuba	1,299,087	11,890	34,226,998	8.6	7.6	C
Curacao	82,604	N/A	N/A	6.9	N/A	C
Christmas Island	994	N/A	N/A	4.2	N/A	SEA
Cayman Islands	47,154	1,318	N/A	6.6	2.7	C
Djibouti	333,054	901	N/A	7.8	2.3	ME
Dominica	35,073	1,377	N/A	6.4	2.8	C
Dominican Republic	790,588	9,000	13,812,145	8.3	7.0	C
Ecuador	3,100	10,439	N/A	4.9	4.8	EP
Egypt	571,170	205,260	32,826,014	8.1	7.9	ME
Eritrea	251,926	11,255	1,744,782	7.6	5.5	ME
Fiji	383,845	43,475	15,703,945	7.9	7.0	GBR,M, P
Federated States of Micronesia	85,748	23,413	198,862	6.9	5.7	M
Guada-loupe	220,058	2,446	3,610,737	7.5	6.0	C
Grenada	42,931	1,953	1,585,918	6.5	5.4	C
Honduras	37,825	12,454	4,959,989	6.4	6.2	C
Haiti	1,475,746	55,045	3,973,142	8.7	6.5	C
Indonesia	12,198,508	1,657,757	107,542,434	10.0	10.0	SEA
India	6,555,868	958,530	274,882,625	9.6	9.4	SEA, CIO
Iran	257,039	15,953	50,506,029	7.6	7.9	ME
Israel	0	400	3,370,972	0	6.0	ME
Jamaica	617,623	20,000	16,599,802	8.1	7.1	C
Jordan	33,519	90	59,792	6.4	3.2	ME
Japan	623,273	30,576	234,793,089	8.1	8.9	SEA
Kenya	521,948	12,938	5,338,532	8.0	6.3	WIO
Kiribati	94,244	14,260	11,241,006	7.0	6.8	CP, M, P
St. Kitts and Nevis	19,664	488	2,156,335	6.0	5.7	C
Kuwait	148,967	3,566	2,541,630	7.3	5.8	ME
St. Lucia	96,101	1,040	192,170	7.0	4.0	C
Sri Lanka	944,093	22,417	4,752,304	8.4	6.2	CIO
Madagascar	833,698	30,000	3,991,132	8.3	6.1	WIO
Maldives	223,017	30,223	990,466	7.5	5.9	CIO
Mexico	425,711	64,705	231,700,594	7.9	8.9	C, EP
Marshall Islands	50,258	21,743	N/A	6.6	5.6	M
Myanmar	180,331	123,746	N/A	7.4	7.4	SEA
Northern Mariana Islands	53,678	603	N/A	6.6	1.9	M
Mozambique	253,243	50,326	126,557	7.6	6.4	WIO
Montserrat	1,715	N/A	N/A	4.5	N/A	C
Martinique	146,793	2,500	5,793,451	7.3	6.4	C
Mauritius	265,262	7,127	18,934,530	7.6	7.2	WIO
Malaysia	1,142,333	83,720	248,586,246	8.5	9.0	SEA
Mayotte	147,666	1,005	8,594	7.3	2.5	WIO
New Caledonia	136,153	23,539	3,542,389	7.2	6.0	GBR
Nicaragua	5,814	6,755	29,463,860	5.3	7.5	C
Niue	827	607	N/A	4.1	1.9	P
Nauru	6,916	292	653	5.4	1.2	M
Oman	314,288	10,287	90,832,869	7.7	8.3	ME
Panama	84,304	6,551	53,387,993	6.9	7.9	C, EP
Philippines	12,963,664	911,754	705,110,034	10.0	9.7	SEA
Palau	13,043	3,795	109,462	5.8	3.8	M
Papua New Guinea	609,016	107,952	2,420,370	8.1	7.2	GBR
Puerto Rico	897,188	1,163	11,208,717	8.4	6.8	C
French Polynesia	221,276	21,495	1,765,467	7.5	5.6	P
Qatar	42,443	4,505	30,795,948	6.5	7.5	ME
Reunion	109,925	1,060	375,123	7.1	4.4	WIO
Saudi Arabia	2,190,247	24,500	132,227,485	8.9	8.5	ME
Sudan	3,555	27,254	50,237	5.0	5.8	ME
Singapore	78,342	1,529	803,050	6.9	5.0	SEA
Solomon Islands	307,616	58,390	N/A	7.7	6.6	GBR
Somalia	176,955	3,694	4,509,732	7.4	6.2	ME, WIO
Sint Maarten	26,959	N/A	N/A	6.2	N/A	C
Seychelles	59,299	2,000	5,485,708	6.7	6.3	WIO
Turks and Caicos Islands	20,480	2,524	38,212,573	6.1	7.7	C
Thailand	233,667	99,807	568,253,338	7.5	9.6	SEA
Tokelau	1,250	179	N/A	4.4	0.7	CP
Timor Leste	97,846	5,415	11,354	7.0	4.2	SEA
Tonga	84,729	7,170	249,913	6.9	4.5	P
Trinidad and Tobago	27,285	6,005	2,335,424	6.2	5.7	C
Tuvalu	9,611	2,708	N/A	5.6	3.5	M
Taiwan	186,430	26,516	100,911,037	7.4	8.3	SEA
United Rep. of Tanzania	1,612,870	108,789	28,586,374	8.7	7.5	WIO
United States	1,983,056	29,596	N/A	8.9	5.9	C, CP
St. Vincent & the Grenadines	42,323	587	33,632	6.5	2.8	C
Venezuela	396,002	21,291	160,788,383	7.9	8.7	C
British Virgin Islands	17,678	1,579	2,682,973	6.0	5.8	C
US Virgin Islands	34,003	981	6,598,431	6.4	6.4	C
Viet Nam	1,581,789	204,546	N/A	8.7	7.9	SEA
Vanuatu	112,666	9,410	67,499	7.1	4.7	GBR
Wallis & Futuna Islands	12,037	10,357	18,776	5.7	4.8	M, P
Samoa	110,024	3,586	704,204	7.1	4.9	P
Yemen	553,291	20,993	106,057,336	8.1	8.4	ME
South Africa	17	N/A	N/A	1.8	N/A	WIO

**Fig 3:**
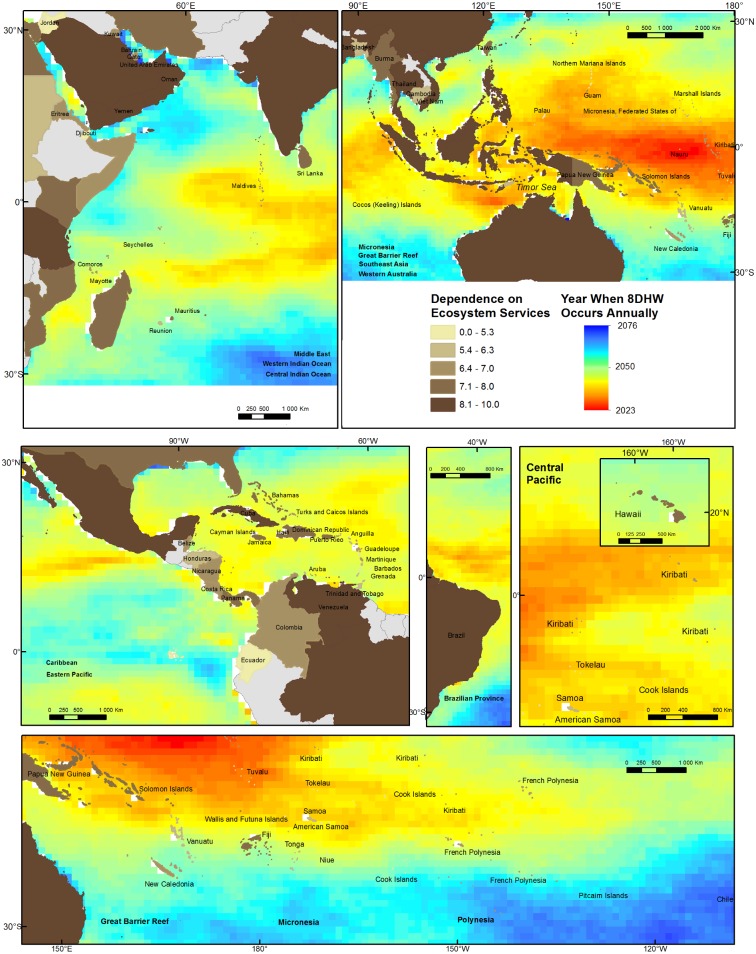
Country-level dependence on coral reef ecosystem services and future risk of coral bleaching. Bleaching risk is indicated by the year when DHW8 is first reached annually, under RCP8.5 scenario [24,25]. Ocean Provinces are indicated in each panel in bold. Earlier years indicate increased bleaching risk.

**Fig 4:**
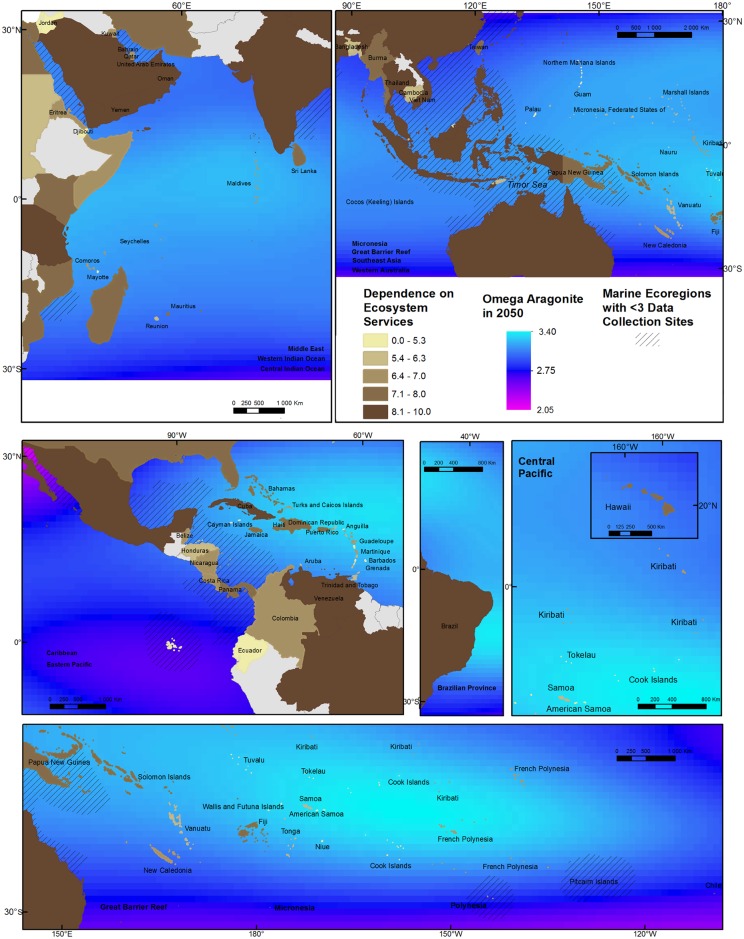
Country-level dependence on coral reef ecosystem services and future risk of ocean acidification as omega aragonite level in 2050 based on GLODAP, CARINA and PACIFICA data, [43]. Ocean Provinces present in each panel in bold. Lower omega aragonite levels reflect higher ocean acidification risk.

To understand where humans may be most exposed to changes in coral reef health caused by increased atmospheric CO_2_, we combine our map of scores for combined human dependence on coral reefs with a) projections that show when sea surface temperature may reach levels that will cause bleaching (Fig 3) and the intensity of OA (Fig 4) in the near future (2050) [43].

Specifically, we use published projections [25] of the year when sea surface temperature is expected to reach 8 DHW on an annual basis. These previously published projections are based on an ensemble of models that are included in the IPCC Fifth Assessment Report (CMIP5) for the emission scenario RCP8.5, OISST V2 1982–2005 climatology [4]. Corals are known to bleach when 6 DHW occur [17]. The annual occurrence of 8 DHW has been cited as a level of thermal stress that will lead to significant coral mortality [24,25]. (Other measures of elevated sea surface temperature could be used, but give the same spatial distribution.) The numerical data for the projected year when 8 DHW are predicted to first occur annually are presented in the supplementary materials (S1 Data). To understand where high thermal stress will put people at risk, we overlay maps of 8 DHW with the indicator scores of human dependence (Fig 3).

OA will affect a number of physiological and even behavioral processes that are important to coral reef ecosystems [7], each affected differently by changes in ocean carbon conditions. As a result, there is no agreed-upon, single threshold that represents when coral reefs will be compromised by OA. So, we do not set a given threshold and map when that threshold will be reached (as we did for bleaching risk). Instead, to understand where the contribution of CO_2_-driven OA to reef conditions may be severe in the future, we map projections of omega aragonite (Ω_ar_) for the year 2050 under the business-as-usual (RCP8.5) emissions scenario [43]. Ω_ar_ is a measure of carbonate chemistry, related to OA, that reflects the level of carbon saturation of ocean water. It is a measure originally intended to reflect the challenge that OA poses for organisms with calcium carbonate skeletons. Lower levels of aragonite indicate more severe OA. To calculate Ω_ar_ in year 2050, we use the same technique provided by [43] based on data from the global data from the sites included in the Global Ocean Data Analysis Project (GLODAP), Carbon in Atlantic Ocean (CARINA), and Pacific Ocean Interior Carbon (PACIFICA) datasets. We calculate projected Ω_ar_ in 2050 using the projected atmospheric *p*CO_2_ and sea surface temperature in 2050 under the RCP8.5 emissions scenario, in-situ total alkalinity (assuming total alkalinity does not change), as well as the salinity, silicate, and phosphate data, as well as calculated surface water *p*CO_2_ from the base year 2000. Data regarding the predicted Ω_ar_ levels for 2050 are available in the supplementary materials (S2 Data).

We use these projected Ω_ar_ levels in 2050 as broadly indicative of OA severity, noting that biological processes on the reef can significantly alter Ω_ar_ up or down relative to the oceanic value [27,44,45] and that bio-regulation of pH in the face of OA is energetically costly for corals [46,47]. As before, to understand where OA risk could most affect people, we overlay projected Ω_ar_ in 2050 with indicator scores for human dependence (Fig 4).

Model projections of OA often extend to cover all waters of the world [4]. These projections, however, can only be verified using existing data to calibrate the correspondence of the projection with current and past conditions. These data are limited to a large set of collection points, which are not distributed across all coral reef areas and thus do not necessarily reflect OA conditions at all coral reefs [43], especially for coastal areas. To show areas where data are scarce, we use hatched areas that represent marine ecoregions as defined by Spalding [48] for which there are fewer than 3 collection sites that are used in current OA projections (Fig 4) [43].

## Results

### Country-level results

Not all coral reef ecosystems or the human communities that depend upon them will experience the same effects as a consequence of a higher-CO_2_ world. First, countries differ substantially in how much they depend on coral reef ecosystems and services (Table 1, Fig 2). Because we focus on country-level dependence on coral reefs, countries with long coastlines that are bordered by coral reefs tend to have higher than average dependence. For instance, Australia, much of Southeast Asia, Brazil, and Mexico all have high combined human dependence scores when both low elevation coastal population and fisheries are considered. It is noteworthy that a number of smaller countries (e.g. Cuba, Kenya, Fiji, and Madagascar) still have high combined dependence scores owing to their long coastlines and the high density of people in coastal areas.

Figs 3 and 4 show the juxtaposition of human dependence with exposure to future sea surface temperature (and thus widespread coral bleaching) and CO_2_-driven OA, respectively. The countries of Oceania are predicted to suffer from mass coral bleaching soonest, followed by the Coral Triangle countries of Southeast Asia and Australia. All of these areas have high human dependence on coral reefs. van Hooidonk et al. [25] show that changing patterns of sea surface temperature and OA differ spatially, particularly by latitude, due to increasing gas solubility (which affects OA) with colder temperature. As a result, the countries most likely to experience severe OA are generally different from those that will experience the earliest onset of coral bleaching. Baja California (Mexico), Japan, China, and southern Australia are projected to be most exposed to future OA partly because they are at the upper and lower latitudinal bounds of coral reef distribution (and thus generally in cooler waters). Countries that span large ranges of latitude will be exposed to a range of future ocean conditions. For instance, the coastline of Australia most at risk from OA (southeast) is different from that most at risk from bleaching (northwest). The Great Barrier Reef spans areas of high future bleaching and OA.

Many of the countries most dependent upon coral reefs are also the countries for which we have the least robust data on OA (hatched areas in Fig 4). Southeast Asia, India, the Coral Triangle, the Western Caribbean, and northern Australia stand out as areas of high human dependence on coral reefs and possibly low confidence in OA projections due to the scarcity of regular OA data collection points [43].

**Fig 5:**
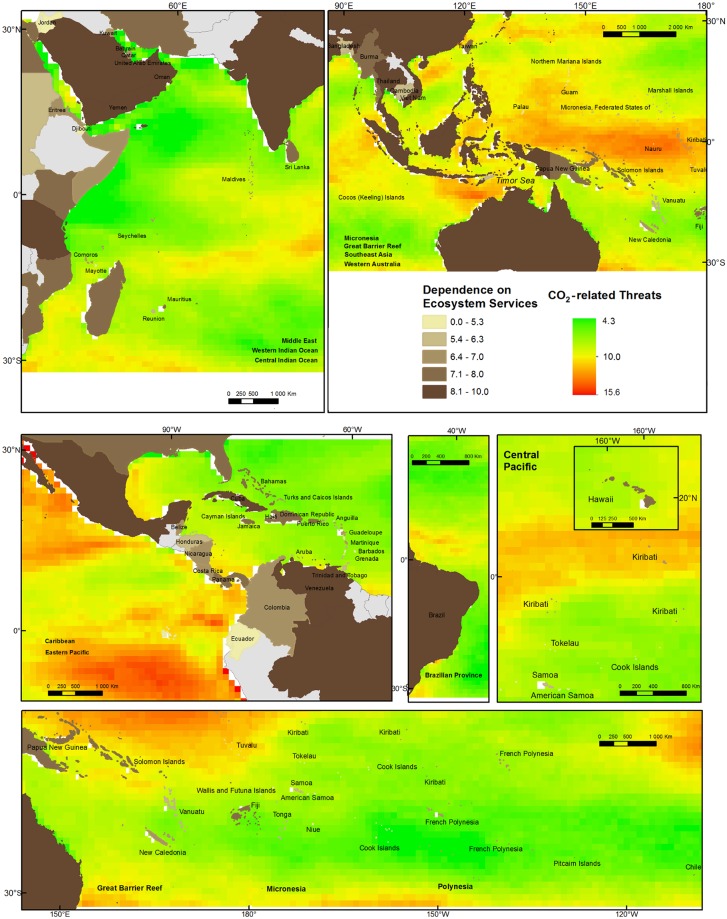
Country-level dependence on coral reef ecosystem services and future combined normalized scores (2–20) for CO_2_-related threats (e.g. ocean acidification and thermal stress). Ocean Provinces are indicated in each panel in bold. Higher scores indicate higher dependence and higher ecological risk.

To understand where coastal communities will face high combined stress from both increased bleaching and more intense OA, we rescale [42] each global threat from 1 (lowest score) to 10 (highest score), sum the normalized scores of both elevated sea surface temperature and Ω_ar_, and map these (Fig 5). Areas that face the highest combined threats from both CO_2_-driven stressors (thermal stress and OA) are highly concentrated, mostly in the Western and Eastern Tropical Pacific. Because OA and increasing sea surface temperature follow different latitudinal gradients [25], no area experiences both the worst possible sea surface temperature and OA conditions (a score of 20). Also, there are no coral reef areas that are completely free from both global stressors (a score of 2) and thus there are no perfect coral reef refuges from the impacts of climate change and OA.

Human dependence on coral reefs and a high combined score for stress from both OA and coral bleaching (e.g. scores approaching 15) are projected to occur along the coasts of much of Western Mexico and Micronesia as well as the coastlines of Indonesia and Australia on the Timor Sea and parts of Southeast Asia. These places may require swift action to protect people from the combined impacts of warming seas and increasingly acidified oceans (e.g. many parts of Southeast Asia).

### Regional results

**Fig 6:**
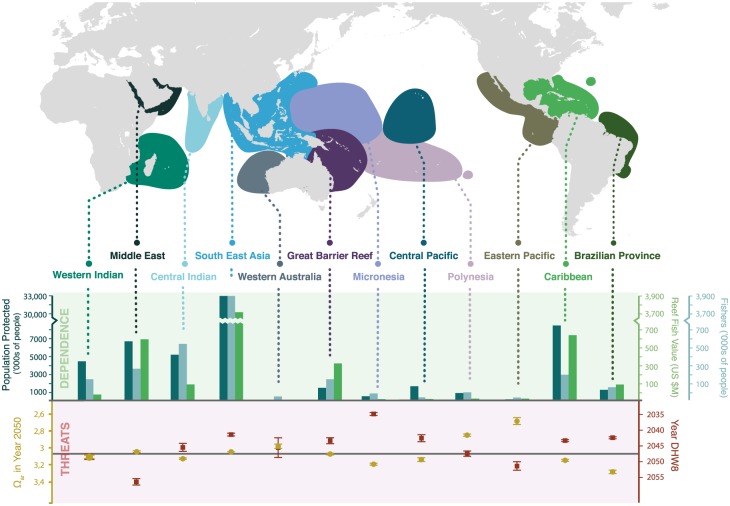
Regional dependence, by ocean province [49], on ecosystem services and average CO_2_-related threats (ocean acidification measured as projected Ω_ar_ levels at coral reefs in 2050 and elevated sea surface temperature as measured by year that 8 DHW are projected to occur annually). The horizontal line in the threats panel represents the mean threat for all regions (scores above this line indicate above average severity of threat). The scales for the reef fish dependence scores are broken to reduce the size of the graph. Note that the Great Barrier Reef Ocean Province includes, but is not limited to, the Great Barrier Reef.

The ecological response of coral reefs is likely to vary regionally due to different species composition, varying rates of change in temperature and acidification conditions, and differences in the conditions that promote coral reef resilience [37] (bottom panel of Fig 6). To visualize the regional threats to coral-reef dependent communities from a high CO_2_ world, we merge coral reef areas into the biological ocean provinces proposed by both Donner [49] and Maina [50]. Within these provinces, we focus only on sea surface temperature (year 8 DHW, S1 Table) and OA (Ω_ar_, S2 table, S3 Data) conditions that spatially co-occur with coral reefs within the province (lower panel Fig 6). Using the same data developed for the country level analysis, we also present province-level results for the total regional human dependence on coral reef ecosystem services (S3 Table, upper panel Fig 6). (Note that when a country’s Exclusive Economic Zone falls within more than one ocean province, we assign human dependence values to each ocean province separately if data were available, e.g. Hawaii and Florida, or we proportionally assign the human dependence data to each province using the same proportion with which reef area was distributed across provinces.) Two of the biological oceanic provinces that will likely face mass, climate-related coral bleaching soonest (e.g. the Micronesian and Brazilian Ocean Provinces) will be exposed to aragonite levels that are less severe than the average across all provinces. Meanwhile, places facing the most severe future OA conditions tend to face a later onset of bleaching (e.g. Middle East). Southeast Asia faces both a rapid projected onset of bleaching (by 2042) and an above average risk from OA (Ω_ar_ = 3.07, which is below the level known to cause reduced growth and recruitment in some corals [30,29]). Southeast Asia also stands out as the biological ocean province that has, by far, the greatest overall dependence on coral reef ecosystem services as measured by total number of people in the region who live at low elevations that are protected by reefs, by number of fishers, and value of fisheries. The Caribbean, the Middle East, and both Indian oceanic provinces also have high human dependence. The Middle East has a high human dependence and faces an above average threat of OA, but a low relative risk of future coral bleaching compared to other regions. The Caribbean faces an above average threat of onset of coral bleaching, but an OA risk that is somewhat below average (note, though, the level of confidence for the Caribbean may be low due to the low number of data collection sites). Similarly, several regions face threats that are above average for one threat and near average for another (e.g. Polynesia and Central Pacific) or near average for both threats including the both Indian Ocean provinces which have high human dependence on coral reefs.

## Discussion

While we are now able to identify many places where coral reefs and the people dependent upon them will be threatened by global environmental changes caused by increased atmospheric CO_2_, we need to do more. Our analysis shows, however, that in many places (see Fig 4) we lack sufficient, routine data collection on OA factors that is needed to identify with confidence the full set of coral reef communities at greatest risk from the combined threats of global environmental change. Areas that have high human dependence and face future stresses from coral bleaching and OA (e.g. Southeast Asia and the Caribbean) also would benefit from data that could better monitor global and local threats to reef health and could be used to design policies to react to reef decline. Also, better human dependence data are needed: data on ecosystem services provided by coral reefs are not regularly collected and no future projections of these data exist.

Model projections about OA in coastal areas, where most coral reefs exist, require data on a large number of local factors (including strong primary production, upwelling, fresh water input, and nutrient overloading [36]). Collecting data everywhere would be infeasible. Not all new data would be equally valuable for decision makers. Therefore, we propose a global strategy, using indicators of human dependence and potential future global climate threats, in order to geographically target areas where new data collection and science will have high social relevance. These are areas where new science and data are needed to inform decision makers about the potential future impacts of bleaching and OA on coral reefs and the people who depend upon them. Similarly, without better data on localized OA conditions, it is difficult to know where future marine protection could most effectively protect potential reef refugia–areas where corals will naturally avoid the stresses of a high CO_2_ world.

The Global Ocean Acidification Observing Network (GOA-ON) was established to better achieve “socially relevant” OA monitoring (Goal 3) [51], and could help focus effort on these areas for better data collection and scientific capacity building. We also need to do more to collect local-level data on the many other environmental stressors that will exacerbate the effects of global environmental change. There are no fully global databases of coral bleaching or the conditions that cause widespread coral mortality.

We focus only on two key stressors associated with increased atmospheric CO_2_, but we recognize that the ecological health of coral reefs depends on many factors [8,35]. Coral death and loss of coral reef cover already is being experienced in many places around the globe (e.g. see estimates of coral reef loss in the Great Barrier Reef [52,53]). Knowing how coral reefs and reef-dependent human communities will fare in a world of rapidly changing global and local environmental conditions will require a better scientific understanding of how combined environmental change affects coral reefs, how coral reef ecosystems may change, and how these reef changes ultimately impact people. Regionally targeted, mesocosm-level or larger field experiments are needed to study the combined effects of global stressors in a way that reflects the regional variation of coral ecology, local human uses, and local environmental stressors.

Finally, we need more and better social and economic science to understand how humans will respond to projected environmental changes in coral reef ecosystems (e.g. the Capturing Coral Reef and related Ecosystems Project, CCRES, project funded by the World Bank and Global Environmental Facility is one such example). New research on human responses to coral reef change is emerging [54–58]. While the literature focuses on the vulnerability and resilience of coral reef ecosystems and the people that depend on them, more empirical study is needed to identify solutions to the socio-economic vulnerability posed by projected changes in coral reef health [59,60]. Human dependence on coral reef ecosystem services is only partially characterized for the present, and rarely projected into the future. The factors that may determine how people will adapt to coral reef decline remain poorly understood [61–63]. Because planning for a high-CO_2_ world has already started, for example through the UNFCCC process [64], science needs to improve fast enough to prevent locking-in approaches that are ineffective or worse. To this aim, empirical research looking at the human responses to ecological changes in coral reefs (e.g. protection, restoration, and socio-economic adaptation planning) and the barriers that impede effective strategies is needed.

To expedite action to combat the changes corals may experience in a high CO_2_ world, new, interdisciplinary science should be conducted in regions where the likely social and economic impacts of bleaching and OA on humans could be high, and thus the potential societal relevance of such science could also be high. Unfortunately, carrying out science and data collection in many of the coral reef regions most at risk of global environmental change is a challenge. Many of these regions lack the financial or human capacity to carry out large-scale experiments and routine data collection. It is often difficult for scientists to obtain permission to sample in coastal ocean areas or where national maritime jurisdictions are disputed. Both international and regional efforts are needed to overcome the impediments to obtaining data in these areas. GOA-ON and other international bodies (e.g. the United Nations Environment Program) should begin to facilitate such cooperation without delay because elevated sea surface temperatures and critical levels of OA are upon us. While reducing atmospheric CO_2_ should remain a primary goal, a portion of international climate change funding that will become available for developing countries in the coming years should go towards supporting this research.

## Supporting Information

S1 Data Data Projections for First Year When 8 Degree Heating Weeks Occur (land areas are masked,[24]). (NC)

S2 Data Data Projections of Ω_ar_ in 2050 (derived from [43]). (NC)

S3 Data Data Projections for First Year When 8 Degree Heating Weeks Occur (land areas are masked, for coral reef areas only [24]). (NC)

S1 Table Oceanic Province Level Data on Sea Surface Temperature (Year When Annual DHW = 8) [24,25]. Derived from an ensemble of models that are included in the IPCC Fifth Assessment Report (CMIP5) for the emission scenario RCP8.5, OISST V2 1982–2005 climatology.
(DOCX)

S2 Table Oceanic Province Level Data on Ocean Acidification in Year 2050 (Omega Aragonite). Based on GLODAP, CARINA and PACIFICA data [43].
(DOCX)

S3 Table Oceanic Province Level Data on Human Dependence on Coral Reef Ecosystems: Fisheries [40] and Low Elevation Coastal Population [41]. (DOCX)
