# Supplementary material for: Coral Reefs and People in a High-CO2 World: Where Can Science Make a Difference to People?
Source: PLoS One. 2016 Nov 9;11(11):e0164699. doi: 10.1371/journal.pone.0164699 (PMC5102364; doi:10.1371/journal.pone.0164699)
Supplement: S1 Table — Derived from an ensemble of models that are included in the IPCC Fifth Assessment Report (CMIP5) for the emission scenario RCP8.5, OISST V2 1982–2005 climatology. (DOCX) [file pone.0164699.s004.docx]

S1 Table. Oceanic Province Level Data on Sea Surface Temperature (Year When Annual DHW = 8) [25,26] derived from an ensemble of models that are included in the IPCC Fifth Assessment Report (CMIP5) for the emission scenario RCP8.5, OISST V2 1982–2005 climatology [45].

| **Ocean Province** | **COUNT** | **MIN** | **MAX** | **Mean Year when DHW8 Occurs Annually** | **STD** |
| --- | --- | --- | --- | --- | --- |
| Brazilian Province | 17 | 2041 | 2045 | 2043 | 0.76 |
| Caribbean | 161 | 2038 | 2055 | 2044 | 2.41 |
| Central Indian Ocean | 45 | 2036 | 2063 | 2046 | 5.72 |
| Central Pacific | 70 | 2031 | 2053 | 2043 | 6.45 |
| Eastern Pacific | 21 | 2043 | 2059 | 2052 | 4.40 |
| Great Barrier Reef | 210 | 2027 | 2068 | 2044 | 9.12 |
| Micronesia | 130 | 2023 | 2042 | 2035 | 4.06 |
| Middle East | 60 | 2040 | 2072 | 2057 | 5.97 |
| Polynesia | 160 | 2036 | 2066 | 2048 | 6.85 |
| South East Asia | 411 | 2030 | 2076 | 2042 | 5.83 |
| Western Australia | 16 | 2033 | 2061 | 2046 | 9.50 |
| Western Indian Ocean | 75 | 2041 | 2058 | 2049 | 4.34 |
