# Supplementary material for: Coral Reefs and People in a High-CO2 World: Where Can Science Make a Difference to People?
Source: PLoS One. 2016 Nov 9;11(11):e0164699. doi: 10.1371/journal.pone.0164699 (PMC5102364; doi:10.1371/journal.pone.0164699)
Supplement: S2 Table — Based on GLODAP, CARINA and PACIFICA data [43]. (DOCX) [file pone.0164699.s005.docx]

S2 Table. Oceanic Province Level Data on Ocean Acidification in Year 2050 (Omega Aragonite) (based on GLODAP, CARINA and PACIFICA data,[43])).

| **Ocean Province** | **COUNT** | **MIN** | **MAX** | **Mean Omega Aragonite** | | **STD** |
| --- | --- | --- | --- | --- | --- | --- |
| Brazilian | 17 | 3.17 | 3.37 | | 3.31 | 0.06 |
| Caribbean | 199 | 2.91 | 3.32 | | 3.17 | 0.11 |
| Central Indian Ocean | 61 | 2.99 | 3.24 | | 3.15 | 0.08 |
| Central Pacific | 71 | 3.00 | 3.39 | | 3.16 | 0.13 |
| Eastern Pacific | 33 | 2.17 | 2.99 | | 2.70 | 0.20 |
| Great Barrier Reef | 223 | 2.44 | 3.24 | | 3.10 | 0.14 |
| Micronesia | 130 | 3.15 | 3.31 | | 3.20 | 0.04 |
| Middle East | 109 | 3.03 | 3.11 | | 3.07 | 0.02 |
| Polynesia | 162 | 2.63 | 3.39 | | 3.23 | 0.14 |
| Southeast Asia | 544 | 2.74 | 3.18 | | 3.07 | 0.08 |
| Western Australia | 43 | 2.59 | 3.13 | | 3.00 | 0.13 |
| Western Indian Ocean | 76 | 2.87 | 3.22 | | 3.13 | 0.09 |
