# Supplementary material for: Coral Reefs and People in a High-CO2 World: Where Can Science Make a Difference to People?
Source: PLoS One. 2016 Nov 9;11(11):e0164699. doi: 10.1371/journal.pone.0164699 (PMC5102364; doi:10.1371/journal.pone.0164699)
Supplement: S3 Table — (DOCX) [file pone.0164699.s006.docx]

S3 Table. Oceanic Province Level Data on Human Dependence on Coral Reef Ecosystems: Fisheries [40] and Low Elevation Coastal Population [41]

| **Ocean Province** | **Fishers**  **(jobs)** | | **Value of catch**  **(US$ 2005)** | **Low Elevation Coastal Population Protected by Coral Reef**  **(# of people)** |
| --- | --- | --- | --- | --- |
| Brazilian Province | | 144,433 | 180,174,864 | 1,239,637 |
| Caribbean | | 276,826 | 726,828,415 | 8,300,897 |
| Central Indian Ocean | | 620,974 | 168,726,685 | 5,054,227 |
| Central Pacific | | 25,495 | 1,811,215 | 1,536,879 |
| Eastern Pacific | | 23,123 | 17,415,220 | 108,616 |
| Great Barrier Reef | | 225,201 | 413,564,715 | 1,441,968 |
| Micronesia | | 72,340 | 11,688,205 | 407,388 |
| Middle East | | 345,455 | 669,087,061 | 6,535,613 |
| Polynesia | | 84,622 | 17,189,967 | 809,403 |
| South East Asia | | 3,971,693 | 3,768,035,428 | 33,187,672 |
| Western Australia | | 2,902 | 45,816,675 | 30,990 |
| Western Indian Ocean | | 228,112 | 66,251,593 | 4,271,981 |
